# Resistance Mechanisms of the Metastatic Tumor Microenvironment to Anti-Angiogenic Therapy

**DOI:** 10.3389/fonc.2022.897927

**Published:** 2022-05-19

**Authors:** Lars M. Schiffmann, Christiane J. Bruns, Thomas Schmidt

**Affiliations:** Department of General, Visceral, Cancer and Transplantation Surgery, Faculty of Medicine and University Hospital Cologne, University of Cologne, Cologne, Germany

**Keywords:** anti-angiogenic therapy, resistance, metastatic microenvironment, angiogenesis, tumor microenvironment

## Abstract

Angiogenesis describes the formation of blood vessels from an existing vascular network. Anti-angiogenic drugs that target tumor blood vessels have become standard of care in many cancer entities. Though very promising results in preclinical evaluation, anti-angiogenic treatments fell short of expectations in clinical trials. Patients develop resistance over time or are primarily refractory to anti-angiogenic therapies similar to conventional chemotherapy. To further improve efficacy and outcome to these therapies, a deeper understanding of mechanisms that mediate resistance to anti-angiogenic therapies is needed. The field has done tremendous efforts to gain knowledge about how tumors engage tumor cell and microenvironmental mechanisms to do so. This review highlights the current state of knowledge with special focus on the metastatic tumor site and potential therapeutic relevance of this understanding from a translational and clinical perspective.

## Introduction

Angiogenesis is a biological process that describes the formation of new blood vessels from an existing vascular network. It is essential in many physiological processes including embryonical development or the female reproductive system (1). It is furthermore highly relevant in many diseases.

When a developing malignant lesion reaches a critical size, diffusion does not sufficiently cover the increased demand for nutrients and oxygen. The core of this lesion becomes hypoxic leading to the stabilization of HIF-1 alpha. This induces the upregulation of many target genes that foster tumor progression. Among them are several so-called pro-angiogenic genes that orchestrate the ‘angiogenic switch’ by which the tumor recruits blood vessels from the surrounding healthy tissues enabling exponential tumor growth (2, 3). Besides this ‘classical’ mode of sprouting angiogenesis tumors engage other mechanisms of vascularization such as intussusceptive angiogenesis or vasculogenic mimicry (4, 5).

Long before tumor angiogenesis was viewed ‘officially’ as one central ‘hallmark of cancer’ that is crucial for tumor progression at the primary tumor site and metastatic dissemination (6–8), Judah Folkman in the 1970s coined the hypothesis that a malignant tumor could be forced to regression by attacking its vasculature (9, 10). Propelled by this postulation many growth factors and signaling pathways that mediate (tumor-) angiogenesis have been discovered and plethora of substances were developed to inhibit or modulate angiogenic cascades in tumors in the following. Studies from Hurwitz and Kabbinavar 2003 and 2004 first demonstrated that Bevacizumab, a monoclonal antibody against vascular endothelial growth factor (VEGF) improved response rates and prolonged survival in patients with metastatic colorectal cancer (11, 12). Accordingly, anti-angiogenic therapies, mainly bevacizumab and recently ramucirumab, an anti-VEGFR2 antibody, have become an important part of many tumor therapies including in colorectal cancer, gastric cancer, renal cell cancer, ovarian cancer and non-small-cell lung cancer (13). Until now most of the clinically approved anti-angiogenic drugs target the VEGF signaling pathway.

Compared to initial prospects which based on very promising experimental basic research and preclinical data (14–16) as well as pivotal clinical trials (11, 12), anti-angiogenic therapies fell short of expectations regarding efficacy, both as a single agent and in combination with chemotherapy. Correspondingly, patients develop resistance towards anti-angiogenic therapies that clinically present in the same way as refractoriness against conventional chemotherapy which occurs during disease progression (17). In this light, one of the major challenges of (tumor)-angiogenesis research is to identify modes of resistance and develop strategies to overcome them.

In parallel to the Hurwitz Trial researchers sought to find prove and insights in how VEGF blockade with bevacizumab exactly works to inhibit tumor growth and progression. It became clear that the main mode of action of pharmacological VEGF withdrawal is the correction of functional and structural tumor blood vessel abnormalities. This has been summarized by Jain under the term ‘vascular normalization’ (18).

It became clear that not only different tumor cell derived pro-angiogenic growth factors contribute to resistance against VEGF blockade, but also tumor stromal cells crucially mediate the efficacy and response to VEGF targeted therapies.

Preclinical evaluation studies in mice exploring the efficacy of anti-angiogenic therapies have mainly been performed in disease models that only partially mimic clinical cancer situations. Many experimental findings are based on subcutaneous tumor models that involve a large primary tumor at best with metastasis at a single organ site and often without metastasis. Clinical evaluation and application of anti-angiogenic therapy, beside very few indications, take place in stage IV situations, often as second- or third-line therapy. This altogether makes preclinical and clinical findings often difficult to compare. Still cancer patients for the most part die from disseminated metastatic disease and anti-angiogenic therapy is mostly used in this disease stage. It is therefore very likely that metastatic lesions and their tumor microenvironment significantly contribute to resistance to anti-angiogenic therapies. This review will give a focused overview over the current state of knowledge of mechanisms of resistance that is mediated by the tumor microenvironment with specific respect to the metastatic tumor site and its potential clinical implications.

## Alternative Pathways

The simplest concept of resistance to VEGF inhibition is the compensatory upregulation of alternative pathways. Accordingly, several dual or multi-targeting approaches that involve mainly the angiopoietin-2 (ANG-2)/TIE2 axis, platelet derived growth factor receptor beta (PDGFR-beta) signaling and fibroblast growth factor receptor (FGFR) signaling (19–25) have been developed and tested preclinically and are e.g. with drugs such as regorafenib or nintedanib clinically approved concepts. Combined VEGF/PDGF signaling blockade has also been tested in a phase I/II trial with promising efficacy and acceptable toxicities, but further clinical studies are lacking until now (26).

### Targeting the Angiopoietin/TIE2 Axis

Targeting or manipulating ANG-2/TIE2 signaling has been demonstrated to show beneficial effects on tumor vascularization, vascular normalization and prolonged survival in murine models of multimodal treatment strategies (27, 28) (29). Clinical studies testing ANG-2/angiopoietin-1/TIE2 inhibition with various substances failed to mirror the promising preclinical results which is presumably due to the complex context-dependent impact of the angiopoietin/TIE2 axis on the endothelium and other tumor stromal cells such as myeloid derived cells (30).

Targeting both VEGF and ANG-2 had additive effects on tumor growth, vascularity and vascular normalization in preclinical models by various mechanisms (13, 15, 17, 23–25). The eagerly awaited McCAVE trial failed to demonstrate a relevant advantage of combined ANG-2 and VEGF blockade with vanucizumab, a dual humanized monoclonal antibody binding both, VEGF and ANG-2, compared to bevacizumab when both drugs were combined with mFOLFOX-6 in previously untreated metastatic colorectal cancer (31). These results were unexpected based on previous trials and have to be further substantiated (32).

One of the perennial questions also here remains how findings from preclinical models that focus on primary tumor growth can be translated into stage IV clinical diseases. One phenomenon highly relevant for systemic cancer disease that seems to be tightly connected to resistance to anti-VEGF therapy that can potentially overcome by ANG-2 inhibition or ANG-2/TIE2 manipulation is the recruitment of myeloid cells to primary tumors and metastatic lesions.

## Tumor-Infiltrating Immune Cells

Tumor-infiltrating myeloid cells constitute the majority of the cellular tumor stroma. They can hinder or foster tumor progression depending on the disease entity, stage and treatment modality, specifically in the context of anti-angiogenic treatment (17, 24, 28–30). Accordingly, with respect to angiogenesis tumor-infiltrating macrophages and neutrophils contribute to resistance to anti-angiogenic therapy in multiple ways (33, 34).

### CD11b^+^ GR1^+^ Cells

**Figure 1:**
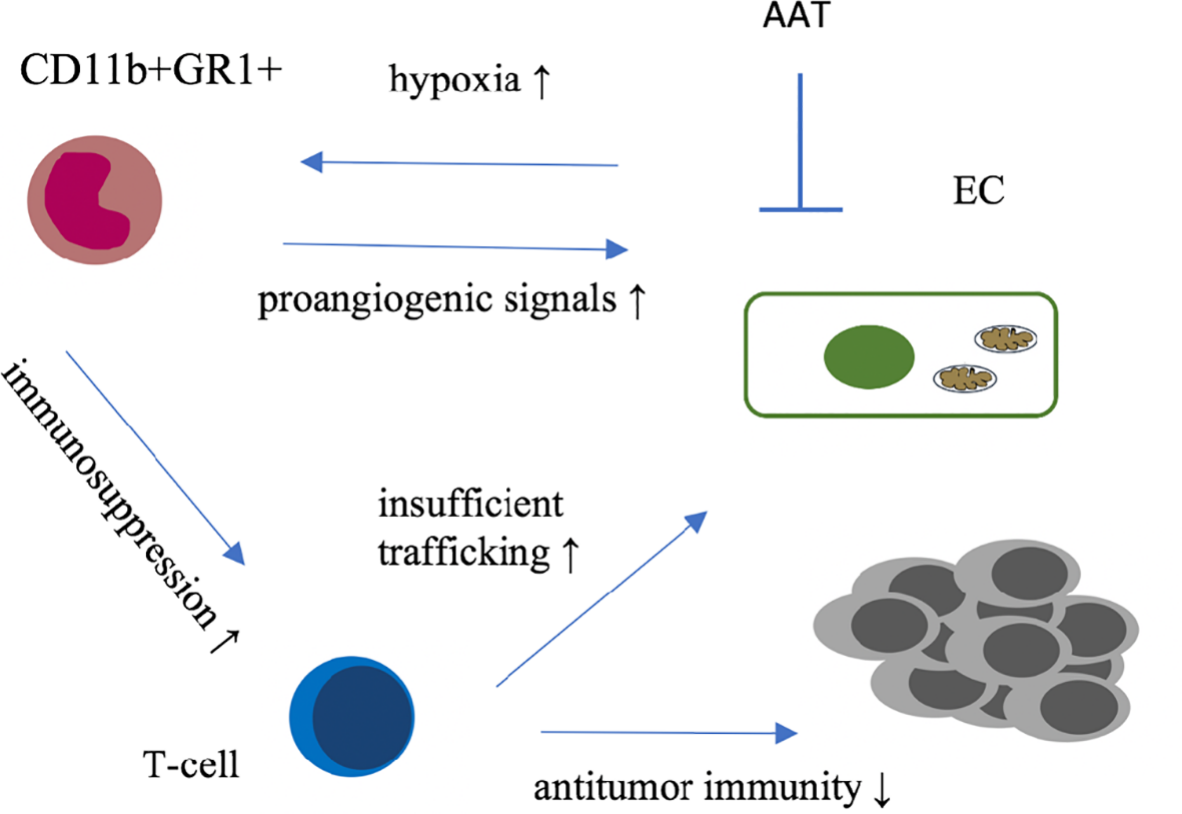
Graphical summary of immune microenvironmental interactions involved in resistance to antiangiogenic therapies. GR1+ cells (i) infiltrate tumors as response to AAT-induced hypoxia and secrete proangiogenic factors; (ii) Furthermore these immune cells are suspected to suppress T-cell activity mitigating anti-tumor immunity. Together with insufficient trafficking of immune cells along structurally and functionally insufficient blood vessels theses mechanisms provide a rational for complementary anti-tumor activity of vascular normalizing AAT and immunotherapy. AAT, antiangiogenic therapy; EC, endothelial cell.

A broad spectrum of neutrophils, macrophages and myeloid-derived suppressor cells (MDSCs) characterized by positivity for CD11b and GR1 (Ly6G/C) have been found to be associated with refractoriness to anti-VEGF therapy in multiple murine tumor models (35). This was at least partially mediated by a cross-talk between granulocyte colony-stimulating factor (G-CSF) and bone-marrow derived Bombina variegate peptide 8 (Bv-8) (33). Targeting Bv-8 with a specific antibody in conjunction with metronomic gemcitabine improved outcomes in a murine model of pancreatic cancer by counteracting pro-angiogenic and pro-metastatic effects of tumor-infiltrating MDSCs (36), compare **Figure 1**.

Whether targeting bone-marrow derived sources of resistance to anti-angiogenic therapy can be translated into the clinic warrants further careful investigation specifically in the context of metastasis.

### Tumor-Infiltrating Macrophages/Neutrophils Primary Tumor Versus Metastasis

The role of tumor-infiltrating neutrophils apparently seems to be divergent depending on the tumor stage. While their occurrence is beneficial at early stages of CRC tumorigenesis (37), increased infiltration of local lymph node or distant organ metastasis with CD177^+^ neutrophils predicted poor outcome to bevacizumab containing chemotherapy in patients with stage IV colorectal cancer (25). Resistance to anti-angiogenic therapy in an anti-VEGF therapy refractory murine model could be overcome by combined inhibition of anti-VEGF and ANG-2 inhibitory treatment (25). There are several potential explanations how ANG-2 blockade can render anti-VEGF treatment induced neutrophil recruitment. First, a specific subset of tumor-infiltrating immune cells express TIE2 (TIE2 expressing monocytes, TEMs) which would directly be targeted by ANG-2 blockade (38). Second, ANG-2 renders the endothelium more sensitive to immune cell binding and infiltration towards the parenchyma/tumor (39, 40).Third, combined anti-VEGF and ANG-2 inhibition enhanced anti-tumor activity of CD8^+^ cytotoxic T-cells and showed complementary effects with immunotherapy (41). Furthermore, blockade of VEGF enhances endothelial adhesion molecules which most likely acts synergistically with the above named mechanisms (42, 43). All mentioned mechanism can be seen as relevant for an unresected primary tumor and for metastatic lesions.

There is ample evidence that VEGF inhibition triggers the recruitment and priming of neutrophils fueling metastasis and progression. An increased neutrophil/lymphocyte ratio predicts outcome of patients with colorectal cancer independent of anti-VEGF treatment (44). VEGF blockade in an experimental model of neutrophil-driven metastasis promoted disease progression (45). Furthermore, increased systemically circulating neutrophils were associated with poor prognosis in patients receiving bevacizumab containing chemotherapy (46). A particular role of metastasis-infiltrating macrophages was recently defined in colorectal cancer metastasis. Proangiogenic VEGFR1^+^ macrophages in colorectal liver metastases predicted survival in patients, which was also true for circulating VEGFR1^+^ monocytes in these individuals (47).

The exact mechanisms how circulating and metastasis infiltrating neutrophils/macrophages promote cancer progression remain to be elucidated, but certainly more studies that discriminate between primary tumor and metastatic site (25, 47) are urgently needed.

### T-Cells/Immunotherapy

Immunotherapy against cancer mostly with immune checkpoint inhibitors (IT) has been integrated into treatment regimens of many cancer entities (48). There is strong evidence that efficacy of the anti-tumor immune response is significantly hampered by specific characteristics of the tumor vasculature and the pro-angiogenic microenvironment. For example, CD8^+^ T-cell infiltration into tumors is disturbed in part due the structural defective and dysfunctional vascular system, T-cell effector functions are manipulated and pro-angiogenic molecules can promote CD8^+^ T-cell exhaustion (49–51) whereas M2-like macrophages and certain subtypes of T-cells secrete proangiogenic factors thereby directly foster tumor angiogenesis (52), see also **Figure 1**.

Accordingly, based on many preclinical studies, combining IT and anti-angiogenic therapy has been suggested as a promising synergistic concept (53). Which patients and to which cost regarding side effects will benefit from combining anti-angiogenics and IT will be deciphered in clinical trials that are currently running for several indications, in general these combinations have already been approved by the FDA (54) (see also **Table 1**). One potential factor that might influence tumor entity specific response to this combination therapy is the sheer abundance of immune cells (e.g. macrophages, T-cells) which differs rigorously between different types of cancer (55).

**Table 1:** overview of some currently recruiting clinical trials investigating anti-angiogenic therapy in conjunction with cancer immunotherapy.

Entity	Interventional arm	NCT number	year of registration
Hepatocellular carcinoma	Ablative therapy* + Bevacizumab + Atezolizumab	NCT04727307	2021
Breast cancer	Paclitaxel + Bevacizumab + Atezolizumab	NCT04732598	2021
Melanoma	Nivolumab+ Axitinb	NCT04493203	2020
Breast Cancer	Paclitaxel + Bevacizumab + Atezolizumab	NCT04408118	2020
Rectal cancer	atezolizumab + bevacizumab	NCT04017455	2019
NSCLC	sintilimab + bevacizumab	NCT04213170	2019

NSCLC, non-small cell lung cancer. * radiofrequency ablation.

## Vessel Co-Option

Tumors do not exclusively engage neoangiogenesis to recruit and hold a vascular system available. Tumor cells can also grow along existing vasculature of the diseased organ without inducing neoangiogenesis, a term called vessel co-option ^38,39^.

Accordingly, the main target for current clinically approved anti-angiogenic therapy is far less relevant as the vasculature is not dependent on VEGF.

Vessels histologically proliferate less and exert an increased pericyte coverage as indicators for a mature, non-activated vascular systems. It is very important to notice that the simplest measure of tumor vascularity, the microvessel density, does not indicate which type of vascularization, angiogenesis or vessel co-option is present in a tumor (56)

### Vessel Co-Option as Challenge to Target Metastatic Vessels

Especially in metastatic lesions vessel co-option is a frequently observed characteristic of tumor progression and a long-suspected cause of resistance to anti-angiogenic therapy (57). The occurrence of vessel co-option was demonstrated for lung metastasis (58), liver metastasis (59) and brain metastasis (60) among others.

Frentzas and colleagues were able to connect histopathological growth patterns of these metastases that involve vessel co-option to poor response to bevacizumab (61). They could demonstrate that nearly half of the examined CRC liver metastases were vascularized by vessel co-option not ‘classical’ angiogenesis and that patients that suffer from metastatic disease which is driven by vessel co-option have a poor histopathological response and particularly detrimental outcome to bevacizumab containing oncologic treatment.

This work furthermore demonstrated that tumor cells require actin-related protein 2/3 complex (Arp2/3) to successfully perform vessel- co-option. Accordingly, knockdown of ARPC3 a subunit of Arp2/3 blocked cancer cell motility thereby inhibiting vessel co-option and re-sensitizing tumors to anti-angiogenic therapy containing cytostatic treatment (61).

Summarizing, vessel co-option might be a major cause why anti-angiogenic treatment is ineffective for example in a large proportion of patients with CRC liver metastases.

Future efforts should focus on two things: (i) to design clinical trials to prospectively prove that response to and outcome after bevacizumab containing chemotherapy depends on histopathological growth patterns involving vessel co-option, (ii) develop treatment strategies that inhibit both vessel co-option and neoangiogenesis, especially in the context of metastatic disease. Furthermore, it is highly relevant to further clarify the role of anti VEGF therapy with bevacizumab or other drugs in multi-modal treatment strategies. The notion that upfront surgery followed by chemotherapy plus bevacizumab improves patients overall survival compared to upfront surgery plus chemotherapy without bevacizumab in patients with metastatic colorectal cancer underscores how relevant this might be (62).

Another challenge is to develop and clinically evaluate techniques that can pre-therapeutically define the histopathological growth pattern which could guide clinical treatment decisions, e.g. in individual multimodal treatment concepts involving chemotherapy +/- targeted therapy prior surgery (e.g. resection of colorectal liver metastasis) or vice versa (63).

## Metabolic Reprogramming of the Tumor Microenvironment

### Endothelial Cell Metabolism

From a metabolic perspective (neo)-angiogenesis is a highly demanding cellular process. Endothelial cells (ECs) that under quiescent, steady state conditions line the inner surface of each blood vessel, maintain their cellular homeostasis under opulent conditions. They consume low amounts of energy while being exposed to the most comprehensive environment, the blood stream. When a growing malignant lesion secretes proangiogenic signaling molecules that activate endothelial cells this relation between supply and demand is completely shifted. The growing vessel, initially mainly constituted by the endothelial sprout, elongates towards a nutrient poor and hypoxic, acidic environment. To execute this challenging task, endothelial cells undergo a ‘metabolic’ switch that involves upregulation of key metabolic pathways. The knowledge of endothelial specific metabolic features is just beginning to be expanded, especially the specific role of tumor endothelial cells. From a clinical perspective endothelial cell metabolism offers many opportunities to explore novel therapeutic targets that might contribute to overcome resistance to growth factor targeted strategies.

### Endothelial Cell Predilection for Glycolysis

Glycolysis is until now the best characterized metabolic pathway in endothelial cells. Specifically, tumor endothelial cells upregulate their glucose metabolism by several mechanisms. This is noteworthy as tumor cells are also considered to use mainly ‘aerobic’ glycolysis as energy resource and to fuel side pathways. Among other things the following: i) tumor ECs upregulate the glucose transporter GLUT-1 (64), ii) tumor ECs directly or indirectly upregulate the expression of rate limiting glycolytic enzymes, e.g. Phosphofructokinase-2/fructose-2,6-bisphosphatase (PFKFB), specifically its isoenzyme PFKFB3 (65) and iii) ECs express high amounts of lactate transporters (e.g. MCT1) (66).

Knockdown of endothelial cell PFKFB3 inhibited vessel sprouting *in vitro* and vivo. The fact that manipulation of the endothelial cell glycolytic metabolism was able to alter endothelial cell sprout differentiation showed the immense role of endothelial cell metabolism that might overrule even growth factor receptor signals (67). This suggested that endothelial cell metabolism as a growth factor independent engine of vessel sprouting and angiogenesis might contribute to resistance to anti-angiogenic therapy (68).

### PFKFB3 as Novel Anti-Angiogenic Target

Indeed, PFKFB3 has then been proven to represent a promising target to reduce pathological angiogenesis in tumors and other diseases (58, 62, 63). Partial genetic or pharmacological inhibition of PFKFB3 was shown to normalize the tumor vasculature and reduce invasiveness in several tumor mouse models. This was accompanied not by reduced tumor growth at the primary tumor site, the conventional read out for efficacy of anti-angiogenic drugs, but by better control of tumor metastasis indication improved vascular normalization. Especially the small molecule compound 3-(3-pyri- dinyl)-1-(4-pyridinyl)-2-propen-1-one (3PO), an inhibitor of PFKFB3 was shown to control metastasis at an intermediate well tolerable dose in preclinical studies (65).

Inhibition of PFKFB3 with 3PO was shown to exert complementary effects with VEGF blockade by bevacizumab in an orthotopic PDX mouse model of glioblastoma (69). This was mediated by a prolonged vascular normalization window and improved delivery of chemotherapy indicating that inhibition of EC glycolysis might contribute to resistance towards antiangiogenic therapy in glioblastoma.

### Role of Endothelial Oxidative Phosphorylation in Tumor Angiogenesis

Based on early pioneer work endothelial cells have long been viewed as similar to cancer cells to exert a ‘warburg-like’ metabolic phenotype (58, 59). This included the presumption that ECs have very few and dysfunctional mitochondria (70).

This has recently been amended as mitochondrial metabolism and oxidative phosphorylation (oxphos) indeed play an important role in activated endothelial cells and are indeed functionate (71, 72).

Manipulating endothelial cell mitochondrial metabolism has broad effects on endothelial cell integrity and function (71, 73–75). Pharmacological targeting of the mitochondrial respiratory chain and genetic ablation of mitochondrial oxidative phosphorylation reduced tumor growth and vascularity in mice. Surprisingly, metastatic dissemination was increased in mice were endothelial cells lacked functional oxphos (71). The genetic approach included a maximum achievable Cre recombination mediated gene deletion. These results are probably comparable to maximum blockade of EC glycolysis from others (76). Dose escalation to higher doses of 3PO showed a higher efficacy regarding tumor growth reduction (in comparison to lower doses) of primary tumors in mice but failed to control metastasis (76). It remains to be elucidated whether this effect is specific for the manipulation of tumor vessel metabolism or a general phenomenon (compare section ‘dosing of anti-angiogenic therapies). It is also possible that manipulation of endothelial cell metabolism whether it is cytosolic glucose metabolism or mitochondrial metabolism induces cellular signaling processes that directly facilitate metastatic dissemination.

### Lactate as Alternative Substrate and Signaling Molecule

**Figure 2:**
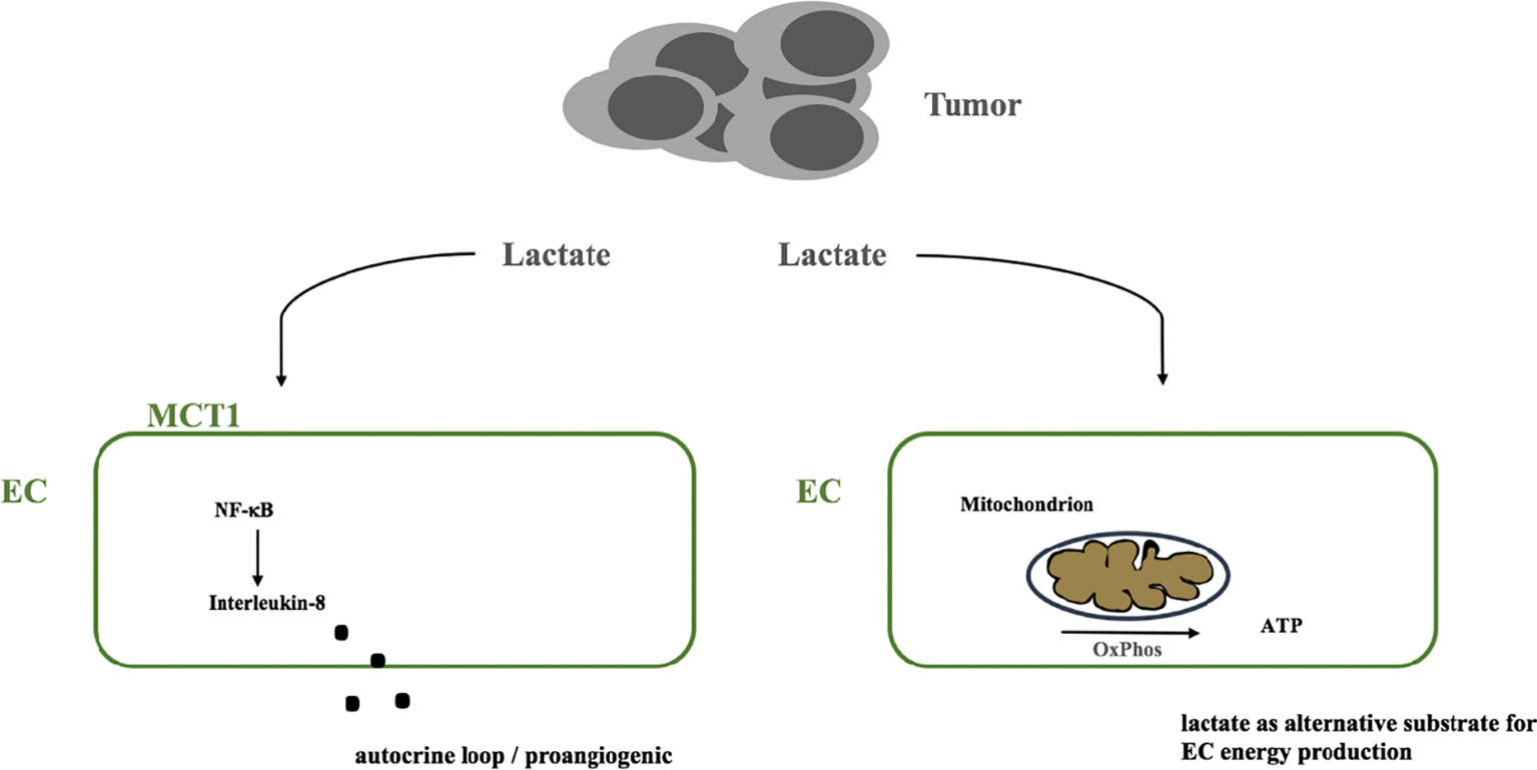
Model of the potential dual role of tumor-derived lactate. (i) as a signaling molecule that triggers a NF-κB dependent autocrine proangiogenic program *via* IL-8 and (ii) as an alternative substrate that ECs metabolize *via* the respiratory chain to produce ATP. MCT1, monocarboxylate transporter 1; EC, endothelial cell; OxPhos, oxidative phosphorylation; ATP, adenosintriphosphate; NF-κB, nuclear factor kappa B.

Potential metabolism related pathways that could contribute to resistance to anti-angiogenic therapy are lactate induced signaling pathways. Endothelial cells were shown to be highly activated by tumor cell derived lactate which induces a NF-κb/Interleukin-8 driven proangiogenic stimulus (77). Beside this lactate induced signaling cues it is possible that ECs take up lactate to metabolize it to pyruvate which is then catabolized *via* the respiratory chain to generate ATP by oxidative phosphorylation (71) a form of metabolic symbiosis similar to processes in the brain (78, 79). Beside the fact that lactate might serve as an alternative substrate in conditions where glucose is scarce, e.g. in the tumor microenvironment, elimination of lactate by endothelial cells might alleviate lactate induced acidity and might limit proangiogenic lactate induced signaling, compare **Figure 2**.

### Endothelial NF-κB and Metastasis

Activated NF-κB signaling in ECs was shown to be associated with poor pericyte coverage. Targeting EC glycolysis reduced NF-κB signaling, tightened EC intercellular junctions and increased pericyte coverage which might in part explain favourable results on metastasis (65).

Interestingly, endothelial specific transgenic mice, that express a ‘superinhibitory’ mutant of iκBα, leading to impaired NF-κB downstream signaling in endothelial cells, showed an impaired endothelial barrier. This resulted in increased metastasis indicating that dysfunctional endothelial NF-κB signaling increases the risk or dynamic of tumor cell dissemination (80). This is not implicitly in contrast to each other, but rather highlights a highly prominent role of endothelial cell NF-κB signaling in cancer progression and metastasis that warrants further investigation. Accordingly, therapeutic approaches that might interfere with EC NF-κB signaling should be carefully designed to modulate overactivation of this pathway without totally inhibit NF-κB related control of endothelial homeostasis. Whether and how direct or indirect targeting of NF-κB signaling in ECs that has been designed to treat inflammatory diseases (81) can be exploited as anti-angiogenic therapies has to be further evaluated.

### Modulation of TAM Metabolism as Therapeutic Opportunity

Another aspect that could contribute to novel pharmacological opportunities to inhibit tumor and stromal metabolism to overcome resistance of the metastatic tumor microenvironment is to gain a deeper understanding of how stromal cells interact with each other on a metabolic level and how tumor cells and stroma cells co-operate to foster tumor progression (compare section above). E.g. tumor associated macrophages (TAMs) can be manipulated towards a hyper-glycolytic metabolic phenotype thereby ‘steeling’ glucose from endothelial cells which results in vascular normalization, lowers hypoxia and decreases metastasis (82). Tumor derived lactate acted as a signaling molecule that polarizes TAMs toward an M2-like differentiation thereby contributing to tumor progression (82, 83). Studies that characterize the metabolic phenotype of TAMs are urgently needed to find out whether and how TAM metabolism contributes to tumor progression, metastasis and resistance to anti-angiogenic therapy.

### Tumor Cell Metabolism and Anti-Angiogenic Treatment

Another aspect is how targeting tumor and stromal metabolism can influence efficacy of anti-angiogenic compounds. Navarro et al. could demonstrate that vascular normalization by anti-angiogenic therapy modulates tumor cell metabolism away from glycolysis towards OxPhos. This sensitized tumor cells to the mitochondrial inhibitor ME344. ME344 acted synergistically with several anti-angiogenic compounds among them regorafenib which showed resistance as a single-agent (84). A phase 0/I trial demonstrated an increased efficacy of ME344 plus bevacizumab compared to bevacizumab as monotherapy in treatment naïve breast cancer (85). Besides the fact that this concept is innovative it is one of the very few trials that demonstrates efficacy of anti-angiogenic/targeted therapies without conventional chemotherapy and beyond in the neoadjuvant setting. Whether and how this concept is effective in metastatic diseases has to be further pursued.

## Dosing of Anti-Angiogenic Therapies

Accumulating evidence suggests that dosing of anti-angiogenic therapy is more complex than previously thought, especially compared to intense multi-substance chemotherapy regimens. Dose escalation of bevacizumab from 5 mg/kg to 10 mg/kg in combination with fluorouracil and leucovorin failed to further improve survival and response to treatment compared to fluorouracil and leucovorin alone in patients with metastatic colorectal cancer. Furthermore, only the lower dose of bevacizumab showed a significant improvement in response rates not the high dose (11).

Reasons for this clinical finding can be multifaceted. Besides biases from the study design and patient recruitment of this study a potential mechanism behind this is related to the window of normalization, in which structural and functional abnormalities of insufficient tumor vessels become corrected improving delivery of chemotherapy. This is dose and time-dependent and varies from tumor entity to entity and potentially from patient to patient making clinical application and patient selection even more complicated (86, 87). Preclinical data strongly support the context that a maximum reduction of both tumor and stromal cell derived VEGF can cause detrimental effects rather than improving cancer outcome (88, 89). Additionally, dose reductions of VEGF inhibition alone or in combination with the inhibition of other pro-angiogenic pathways demonstrated to be superior to higher doses, e.g. in terms of hypoxia (22, 23).

Prior to the introduction of novel anti-angiogenic treatments to clinical application, lessons learned regarding the importance of dosing of anti-angiogenic therapies should be considered.

## ECM Components of the Tumor Stroma

### Empty Basement Membrane Sleeves

Another important question with high clinical relevance is how the (metastatic) tumor reacts on a therapy pause due to drug intolerance, scheduled drug holiday or prior surgery. In several murine tumor models, both murine orthotopic and subcutaneous models, intense VEGF withdrawal eliminates the endothelial compartment of a tumor blood vessel but spares vascular support structures, e.g. the basement membrane and pericytes (14, 82, 83). Following interruption of VEGF blockade endothelial cells rapidly regrow into these scaffolds (90).

Besides of tumor cells and stromal cells solid tumors are composed of extracellular matrix (ECM).

It was observed that VEGF blockade induces the deposition of extracellular matrix (ECM) consisting of collagen I and IV, hyaluronic acid and glycosaminoglycans. This is the case in both murine primary tumors, murine and human metastasis (22, 91). Constant deposition of these ECM components over time contributes to an increased stiffness within tumors. This contributes to therapy resistance by several proposed mechanisms. The increased intratumoral mechanical force compresses tumor blood vessels which hinders delivery of cytostatic therapy (92–94).

### ECM Deposition in Response to VEGF Inhibition in Mice and Humans

Desmoplastic stromal compositions are known to be associated with poor patient outcome e.g. in pancreatic cancer independent of anti-angiogenic therapy (95), it is therefore particular detrimental that VEGF inhibition might even exacerbate this situation and potentially contributes to primary resistance of anti-angiogenic drugs in several cancer entities. A potential strategy to neutralize deposition of extracellular matrix as a response to VEGF inhibition in colorectal cancer liver metastases has been proposed in murine tumor models. Additional therapy with polyethylene glycol conjugated (PEG) hyaluronidase in combination with VEGF inhibition led to a significant reduction of hyaluronic acid in murine colorectal liver metastases compared VEGF blockade as monotherapy (91). Combination treatment of B20.4-1.1, a monoclonal VEGF neutralizing antibody, and PEG- hyaluronidase significantly improved tumor tissue perfusion with Hoechst 33342, a surrogate marker for delivery of cytostatic therapy compared to B20 alone. Furthermore, the combination therapy in conjunction with 5-FU significantly prolonged mice survival compared to B20 alone.

To summarize, deposition of excessive amounts of extracellular matrix components as response to anti-VEGF therapy might represent a targetable mechanism of acquired resistance of the metastatic microenvironment which warrants further investigation.

## Stiffness/Metastasis-Associated Fibroblasts

**Figure 3:**
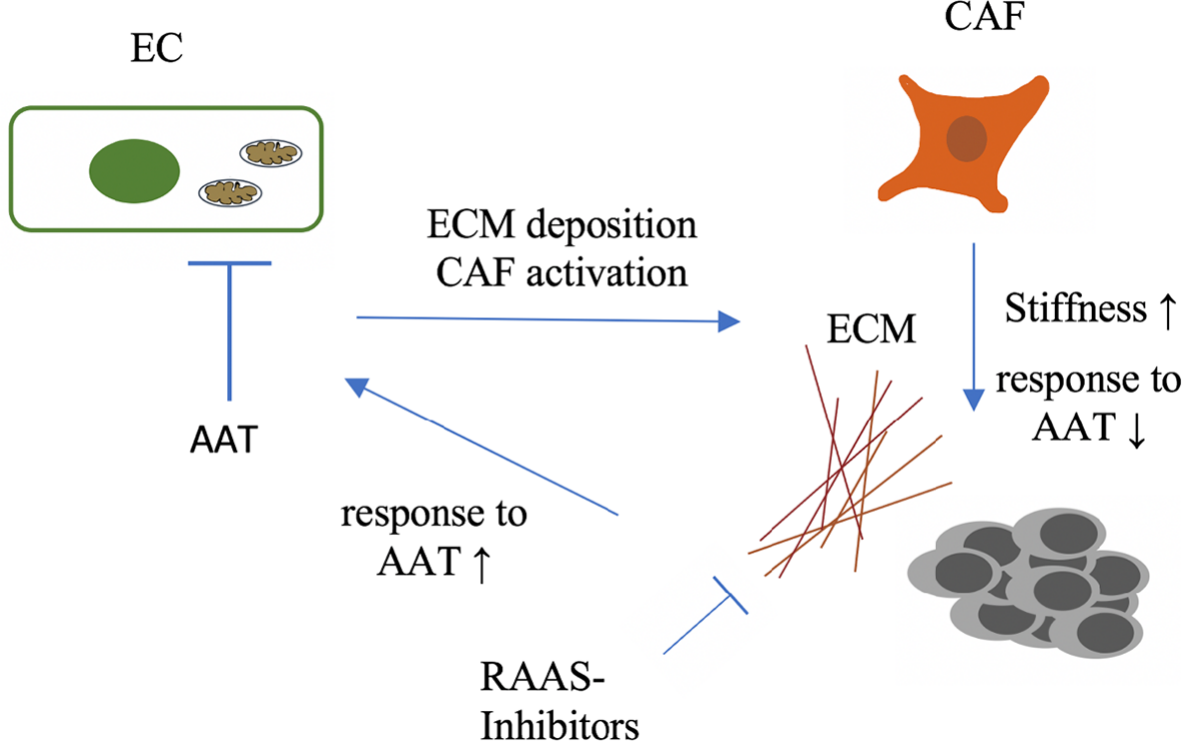
Complex interactions between endothelium and cellular and non-cellular components of the extracellular tumor matrix. Increased tumor stiffness which is in part provoked by AAT itself leads to poor response to AAT and chemotherapy. Tumor stiffness can be targeted by inhibitors of the RAAS, sensitizing patients to therapy. EC, Endothelium; ECM, extracellular matrix; CAF, cancer-associated fibroblast; AAT, anti-angiogenic therapy; RAAS, renin-angiotensin-aldosteron-system.

Primary tumors and metastasis are composed of tumor cells and stromal cells and a considerable amount of extracellular matrix (ECM). Structurally and functionally this tumor ECM composes basement membranes of mainly tumor blood vessels and the ECM of the interstitium (96). The latter besides mechanical and secretory functions that are comparable to healthy organs, significantly contributes to cancer disease progression (97) by several mechanisms. Besides storing growth factors (97) and serving as migration scaffold for several cell types, the ECM contributes to a mechanical phenomenon called tumor stiffness. Stiffness is defined as the capacity of a tissue to resist mechanical force and is composed in tumors mainly by the ECM. Increased tumor stiffness has been identified as a prognostic factor correlated with poor prognosis in several cancer entities (98). A significant determinant of stiffness in tumors is the activation state of cancer associated fibroblasts (CAFs). Activated CAFs induce a constant production of extracellular matrix components such as collagen I and fibronectin, growth factors and employ contractile forces that transforms tissue composition to increase stiffness (92, 93). Increased tissue stiffness has long been considered as resistance factor for anti-angiogenic therapy. Specifically, a role as resistance factor for efficacy of anti-angiogenic therapy in metastasis has recently confined by Shen et al. They could demonstrate that colorectal cancer liver metastases (CRCLM) show a significantly higher rate of stiffness than primary colorectal tumors. Increased stiffness was mainly driven by activation of metastasis associated fibroblasts (MAFs). These MAFs together with the non-cellular tumor stroma composed a proangiogenic microenvironment. MAF activation and stiffness could be targeted by inhibitors of the renin-angiotensin-system (RAS). In CRCLM pharmacological targeting of metastasis stiffness with RAAS inhibitors produced favorable outcomes in conjunction with bevacizumab and chemotherapy compared to chemotherapy and bevacizumab alone (99), compare **Figure 3**. These findings elaborate a mode of resistance against anti-angiogenic therapy specifically for the metastatic environment and suggest a potent and already clinically approved strategy to overcome this mode (100).

### YAP/TAZ as Multi-Faceted Approach to Overcome Resistance

Another very interesting aspect is the role of endothelial YAP (Yes-associated protein) and TAZ (transcriptional coactivator with PDZ-binding motif) which are important regulators of vascular development (101, 102) and are controlled by VEGF and also by mechanical signals (103). Accordingly, YAP/TAZ is involved in both, signaling of the therapeutic target and a potent resistance mechanism of anti-angiogenic therapy in metastatic disease (99). It was recently shown that genetic and pharmacological targeting of endothelial YAP/TAZ inhibits primary colorectal cancer tumor growth in mice. YAP/TAZ nuclear localization was induced by VEGF and TNF and could be inhibited by Verteporfin, a YAP/TAZ inhibitor, in a STAT3 dependent manner (104). Whether pharmacological YAP/TAZ manipulation (105) with verteporfin can be exploited to render (also metastatic) resistance to anti-angiogenic therapy has to be further explored.

## Discussion

Anti-angiogenic therapies have become part of many mostly palliative treatment regimens. After very successful preclinical work and promising first clinical trials 20 years ago, anti-angiogenic therapies failed to revolutionize anti-cancer therapies. Resistance appears after time similar to conventional cytostatic drugs. Tremendous efforts have been performed to uncover potential mechanisms of resistance to anti-angiogenic therapies. Though still nearly two decades after clinical approval of bevacizumab, targeting VEGF is the only broadly clinically applied antiangiogenic concept, not only in colorectal cancer.

One major burden in the development of first-generation anti-angiogenic therapy was to disregard several initially already evident facts: (i) subcutaneous murine tumor models are very different to polytopic metastasized human cancers (ii) vessel co-option is insufficiently targetable with VEGF inhibition (iii) though VEGF is a very potent proangiogenic factor many other cytokines can drive angiogenesis instead (iv) the complex microenvironment(s) of polytopic metastasized cancer diseases exploits a plethora of mechanisms to foster tumor progression independent of VEGF.

Accordingly, future studies should engage models that involve metastasis and test their hypothesis in (ideally) large human cohorts. The field has to balance a difficult bargain between two challenges: first, to bring novel strategies that apparently are more effective than ‘just’ inhibiting VEGF quickly to clinical application, among them combined VEGF and ANG-2 blockade or novel metabolism targeted strategies such as PFKFB3 inhibition; and second, to exclude as best as possible that these interventions produce detrimental unwanted modulations of the tumor and its microenvironment that exhaust the beneficial effects that were pronounced in preclinical studies. This has the potential to further improve patients’ outcome in colorectal cancer, brain cancer, ovarian cancer, esophagogastric cancer and many other entities (106).

Additionally, serum biomarkers and radiologic tools, e.g. image guided determination of the vascular normalization windows (107) are urgently needed to be able to pre-select patients. This would spare unnecessary or even harmful treatments for individuals and uncountable costs for health care systems.

## Author Contributions

All three authors conceptualized and wrote the paper. All authors contributed to the article and approved the submitted version.

## Conflict of Interest

The authors declare that the research was conducted in the absence of any commercial or financial relationships that could be construed as a potential conflict of interest.

## Publisher’s Note

All claims expressed in this article are solely those of the authors and do not necessarily represent those of their affiliated organizations, or those of the publisher, the editors and the reviewers. Any product that may be evaluated in this article, or claim that may be made by its manufacturer, is not guaranteed or endorsed by the publisher.
